# Stability and Recovery of Palytoxin and Ovatoxin‑a

**DOI:** 10.1021/acsomega.5c05500

**Published:** 2025-11-04

**Authors:** Elizabeth M. Mudge, Christopher O. Miles, Valentina Miele, Carmela Dell’Aversano, Pearse McCarron

**Affiliations:** † Metrology Research Centre, 103207 National Research Council of Canada, 1411 Oxford Street, Halifax, Nova Scotia B3H 3Z1 Canada; ‡ Norwegian Veterinary Institute, P.O. Box 64, Ås 1431 Norway; § 60240 University of Napoli Federico II, School of Medicine and Surgery, Department of Pharmacy, Via D. Montesano 49 Napoli 80131, Italy

## Abstract

Palytoxin and ovatoxins belong to a class of marine toxins identified in soft corals and microalgae, *Palythoa* spp. and *Ostreopsis* spp., respectively. Several documented events have resulted in human exposure to aerosolized toxins that led to significant respiratory distress. It has been reported that processing of samples containing palytoxin and ovatoxin during analysis can lead to significant analyte recovery issues due to a variety of parameters. In this study, systematically designed experiments, monitored by LC–MS/MS, were used to evaluate palytoxin and ovatoxin-a stability and recovery, and the effects of pH, solvent composition, and vial contact surface. Significant losses of palytoxin and ovatoxin-a were observed when drying highly aqueous solutions in glass, which were reduced with the use of a polypropylene contact surface and the addition of bovine serum albumin and phosphate-buffered saline. The results showed that palytoxin analogues should be maintained in solutions containing greater than 50% organic solvent, such as methanol, and in a pH range of 5–8 in order to minimize losses or degradation. The recovery of ovatoxin-a was lower than for palytoxin in several experiments, indicating that the structural differences between these analogues may affect solubility or stability. This work provides insight into palytoxin and ovatoxin-a handling, and will help improve analytical measurements, handling during toxicology studies, and minimize losses during isolation protocols for the development of reference materials.

## Graphical abstract

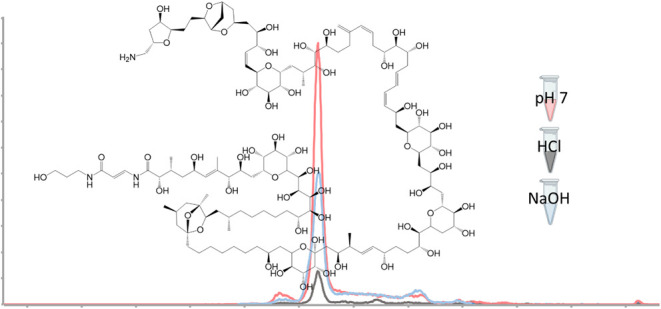

## 1 Introduction

Palytoxins are a class of complex marine toxins originally isolated from zoanthids belonging to the genus *Palythoa*.^1–3^ Additional structurally related analogues, including ovatoxins, ostreocins, and mascarenotoxins, have been detected in a variety of microalgae in the genus *Ostreopsis*, including *O*. cf. *ovata*, *O. fattorussoi*, *O. siamensis*, and *O. mascarenensis*.^4–8^ Palytoxin has been implicated in human toxin exposure events ranging from ingestion of contaminated seafood, inhalation of aerosolized toxins, and cutaneous contact,^9–13^ as well as dog poisonings from consumption of aquarium coral.^14^ Another common form of toxin exposure occurs from improper cleaning and handling of aquarium soft corals, leading to inhalation of aerosolized palytoxins.^13,15–17^ A major concern in Europe is the reoccurring blooms of *Ostreopsis* along the shorelines of the Mediterranean and Adriatic Seas.^11,18^ These blooms have led to significant numbers of individuals being exposed to aerosols containing ovatoxins, leading to respiratory distress.^19^

Palytoxins are large (>2500 Da), amphiphilic natural compounds. The most common features include a conjugated acrylamide–enamide system, a terminal amine, a bicyclic ketal and hemiacetal, two conjugated dienes, several ether rings, and a very high degree of hydroxylation (Figure 1).^20^ In total, over 25 analogues have been reported, varying by degrees of methylation, hydroxylation, or slight modification of their structural backbones. However, many analogues remain only tentatively identified due to their low relative abundance in microalgae, complex structures, and extensive isolation protocols.

**Figure 1:**
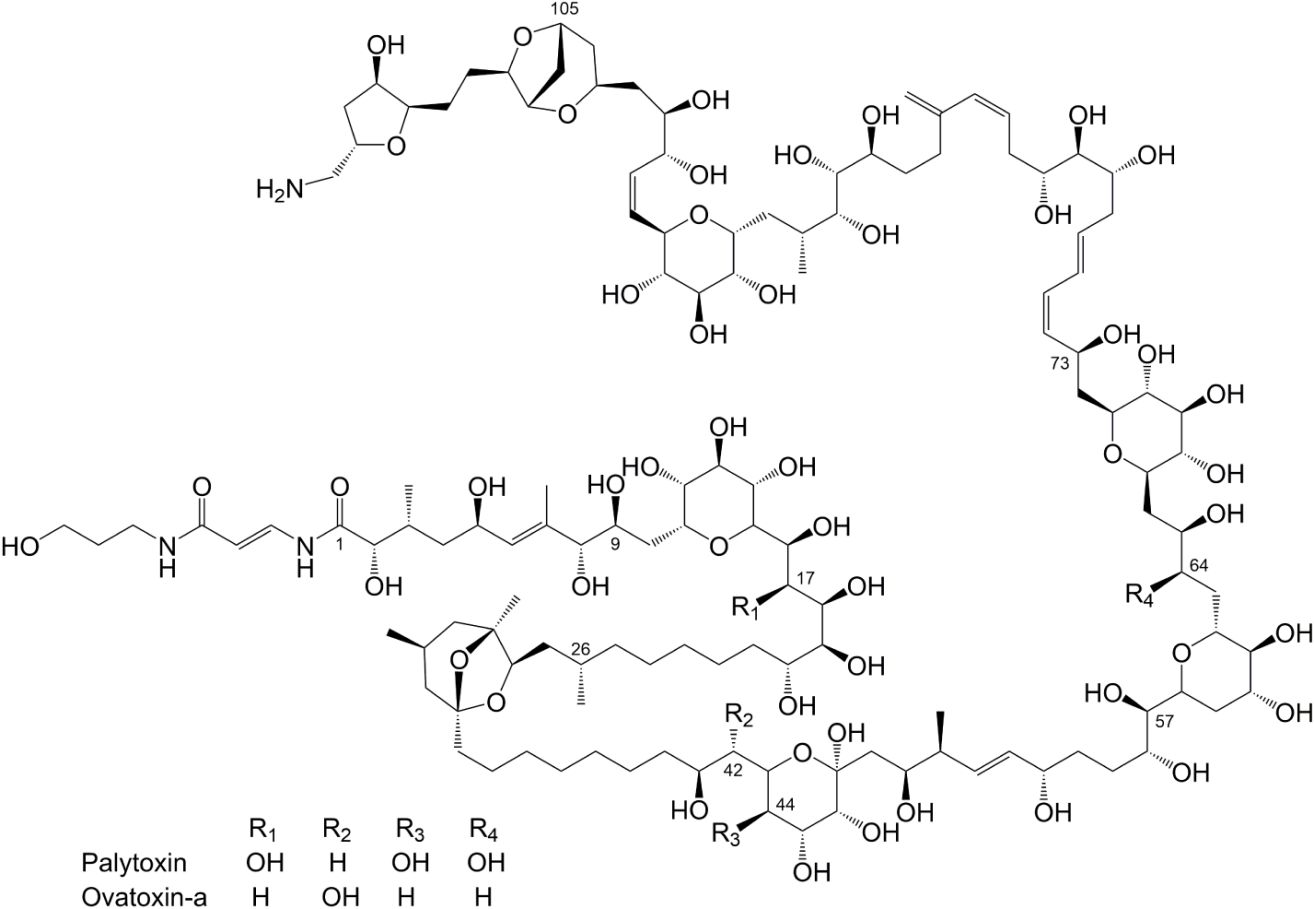
Structures of palytoxin and ovatoxin-a; the stereochemistry of palytoxin is represented, with ovatoxin-a having inverted stereochemistry at C-9, C-26, and C-57.

Palytoxins are known to interfere with the Na^+^/K^+^-ATPase pump, leading to cell membrane depolarization and ionic imbalance impacting cellular function.^20–22^ Based on intraperitoneal mouse bioassays, the relative toxicities of palytoxin, 42-hydroxypalytoxin, and ostreocin D have been shown to be similar,^23,24^ while the limited quantities of other isolated analogues have hampered additional comparisons.^25^ Aerosolized palytoxin, ovatoxin-a and 42-hydroxypalytoxin were extremely potent (LD_50_ values of 0.03–0.05 μg/kg) in a rat model, and relative toxicities were similar for the three analogues.^26^

Mazzeo et al. (2021)^27^ described the difficulties associated with the recovery and isolation of palytoxin, which have significantly hampered both toxicity testing and the development of analytical measurements for this toxin. They reported significant effects of solvent composition, contact surface, and evaporation technique on the recovery of palytoxin.^27^ These effects pose serious problems that require further investigation to enable the acceptable recovery of these complex compounds. The limited availability of reference materials for palytoxin and other analogues also warrants further research on their handling and stability.

The work reported here describes a systematic series of experiments to evaluate the stability, solubility, and recovery of purified palytoxin and ovatoxin-a in order to improve analytical measurement capabilities and isolation protocols. This is a prerequisite for the development of traceable reference materials and validated analytical methods for this class of toxins.

## 2 Materials and Methods

### 2.1 Reagents

LC–MS grade MeCN, MeOH, acetic acid, and formic acid were from Fisher Scientific (Ottawa, ON, Canada). Reagent grade bovine serum albumin (BSA), disodium phosphate, potassium dihydrogen phosphate, ammonium bicarbonate, potassium chloride, sodium hydroxide, hydrochloric acid, and sodium chloride were from Millipore–Sigma (Oakville, ON, Canada). D_2_O and MeOD were from Cambridge Isotope Laboratories (Tewksbury, MA, USA). Distilled water was ultrapurified to 18.2 MΩ·cm using a Milli-Q water purification system (Millipore–Sigma, Billerica, MA, USA). The palytoxin standard (>90% purity by HPLC) was from Wako (Fujifilm Wako Chemicals USA Corporation, Richmond, VA, USA). Ovatoxin-a was isolated from *O*. cf. *ovata*.^28^ HPLC vials and inserts (glass and polypropylene) were from Calibre Scientific (Peterborough, Ontario); polypropylene microcentrifuge tubes were from Fisher Scientific (Mississauga, Ontario); and polypropylene vials were from Waters (Milford, Massachusetts).

### 2.2 Palytoxin and Ovatoxin-a Solutions

Three vials of palytoxin (3 × 100 μg) were combined in approximately 600 μL of 1:1 D_2_O–MeOD and transferred to a 5 mm NMR tube. Quantitative NMR (qNMR) acquisition was performed on a 500 MHz NMR (Bruker, Billerica, MA, USA) according to the method of Burton et al. (2005).^29^ The resonance at 7.79 ppm was used for quantitation of palytoxin using caffeine (TraceCERT; 4.01 mM in D_2_O) as an external calibration standard. The concentration was determined to be 217 μg/mL. The stock solution was diluted to 13.4 μg/mL with 1:1 MeOH–H_2_O and transferred into argon-purged amber glass ampoules and heat-sealed for long-term storage at −80 °C. A working stock solution was diluted to 1.34 μg/mL with 1:1 MeOH–H_2_O and stored at −20 °C.

A stock solution of ovatoxin-a was provided in 1:1 EtOH–H_2_O at approximately 88.4 μg/mL. The concentration was estimated as describe by Miele et al. (2024).^28^ A working stock solution was prepared at 1.77 μg/mL by diluting the stock solution 50-fold with 1:1 MeOH–H_2_O, and it was stored at −20 °C for the subsequent studies (Section 2.3).

### 2.3 Stability Evaluations

#### 2.3.1 pH Stability

The following buffers were prepared (100 mM): phosphate (pH 2, 6, 7, 8), formate (pH 3, 4), acetate (pH 5), and ammonium bicarbonate (pH 9, 10). Strong acid and base conditions were assessed using 100 mM HCl (pH 1) and 100 mM NaOH (pH 13). Palytoxin (268 ng/mL; 100 μL) and ovatoxin-a (177 ng/mL; 100 μL) solutions were prepared by diluting the stock solutions prepared in Section 2.2 with 1:1 MeOH–buffer and stored at ambient temperature (18–22 °C) in nonsilanized glass vials. Palytoxin and ovatoxin-a peak areas were monitored for 24–48 h and plotted versus time to evaluate pH stability. Peak area versus time plots were used to determine the half-lives of palytoxin and ovatoxin-a under unstable conditions.

#### 2.3.2 Solvent Composition

Using the buffers prepared in Section 2.3.1, palytoxin (268 ng/mL; 100 μL) was diluted to 1:9 MeOH–buffer and stored at ambient temperature in nonsilanized glass vials. The palytoxin peak area was monitored over 48 h and plotted versus time to establish pH stability and half-life under unstable conditions.

Palytoxin (335 ng/mL; 500 μL) and ovatoxin-a (177 ng/mL; 100 μL) solutions were each diluted in MeOH–H_2_O from 0 to 100% MeOH, in 10% MeOH increments. The samples were vortex-mixed in polypropylene microcentrifuge tubes and transferred to polypropylene inserts for analysis.

A second set of samples was prepared by combining palytoxin (335 ng/mL) and ovatoxin-a (354 ng/mL) together in MeOH–H_2_O from 0 to 100% MeOH, in 10% MeOH increments (100 μL). The samples were vortex-mixed in polypropylene microcentrifuge tubes. An aliquot (40 μL) was taken immediately and transferred to polypropylene inserts for analysis. The remaining solution was centrifuged at 21,130 *g* for 10 min. A second aliquot (40 μL) was then removed and transferred to a polypropylene insert for analysis.

Triplicate solutions of palytoxin (670 ng/mL) and ovatoxin (704 ng/mL) were prepared separately by diluting with water to 1:9 MeOH–H_2_O in polypropylene microcentrifuge tubes and vortex-mixed. The solutions were allowed to stand at ambient temperature for 1 h, followed by centrifugation at 21,130 *g* for 20 min. The aqueous phase was carefully removed, and 80% MeOH (100 μL) was added to the tubes. The tubes were vortex-mixed and allowed to stand at ambient temperature for 1 h. The solutions were vortex-mixed a second time and then transferred to polypropylene inserts for analysis. These were compared against a control prepared in 80% MeOH at the same concentration.

Triplicate solutions of palytoxin (268 ng/mL, 50 μL) and ovatoxin-a (354 ng/mL; 50 μL) were diluted to 1:9 MeOH–H_2_O in polypropylene vial inserts containing the following additives in the water: no additive; 1 mg/mL BSA; PBS with 1 mg/mL BSA; PBS; 100 mM phosphate buffer (pH 7) with 1 mg/mL BSA; 100 mM phosphate buffer pH 7; 3.5% NaCl with 1 mg/mL BSA; and 3.5% NaCl. These were compared against a control in 1:1 MeOH–H_2_O at the same concentration.

### 2.4 Evaporation

Palytoxin (250 ng/mL; 5 mL) was diluted with 1:1 MeOH–H_2_O, 1:9 MeOH–H_2_O, or 1:9 MeOH–PBS containing 1 mg/mL BSA in triplicate in polypropylene tubes. Following vortex mixing, aliquots (350 μL) were transferred to polypropylene and amber glass vials. Control samples in each solvent and for each contact surface were analyzed directly. Triplicates of the remaining samples were separated into three groups and evaporated to dryness either under a stream of N_2_ at 40 °C, using a vacuum evaporator (Savant SPD2010, SpeedVac Concentrator) at 45 °C, or diluted to 90% H_2_O (if necessary), frozen, and freeze-dried at ambient temperature. The residues were redissolved in 1:1 MeOH–H_2_O (350 μL).

Triplicate palytoxin control solutions (268 ng/mL, 50 μL) were diluted in 1:1 MeOH–H_2_O or 1:9 MeOH–PBS with 1 mg/mL BSA and vortex-mixed. Three additional triplicate sets of samples (268 ng/mL, 50 μL) were prepared in polypropylene vials, with two sets diluted in 1:9 MeOH–PBS containing 1 mg/mL BSA and the third in 1:1 MeOH–H_2_O. All replicates were evaporated to dryness under a stream of N_2_. The replicates from the 1:1 MeOH–H_2_O set were reconstituted in 1:1 MeOH–H_2_O (50 μL). One set from 1:9 MeOH–PBS was reconstituted in H_2_O (50 μL), and the other was reconstituted in 1:1 MeOH–H_2_O.

### 2.5 LC–MS/MS Analysis

All sample preparations were analyzed for palytoxin and ovatoxin-a content using an Agilent 1290 LC equipped with a binary pump, temperature-controlled autosampler, and column compartment, coupled with an Agilent 6495B triple-quadrupole mass spectrometer (Agilent Technologies) equipped with a jet-stream ESI source. Chromatographic separation was performed on a Phenomenex Kinetex C18 column (100 × 2.1 mm, 2.6 μm) using gradient elution. The mobile phases consisted of (A) H_2_O and (B) 95:5 acetonitrile–H_2_O, each containing 30 mM acetic acid. The elution gradient (0.4 mL/min) was: 0–7 min: 23–32% B, 7–7.5 min: 32–100% B, 7.5–10 min: 100% B, with a 2.5 min re-equilibration. The autosampler and column compartments were maintained at 25 °C. The injection volume was 5 μL.

The mass spectrometer and source conditions were optimized using the palytoxin RM, with the gas temperature and flow set to 180 °C and 11 L/min. The nebulizer was 50 psi with a sheath gas temperature and flow rate of 200 °C and 10 L/min, respectively. The capillary voltage was 5.0 kV, and the nozzle voltage was 2.0 kV. The ion funnel low RF was set to 170 and the high RF was 110. The most dominant selected reaction monitoring transitions of the doubly charged ions in positive mode were used: *m*/*z* 1332 [M+2H–H_2_O]^2+^ → 327 for palytoxin and *m*/*z* 1324 [M+2H]^2+^ → 327 for ovatoxin-a, with a collision energy of 22 eV and a cell accelerator voltage of 1 (Figure S1). Peak integration was performed manually with a maximum peak width of 0.4 min, bracketing the primary peak for both compounds in order to ensure consistent baseline integration and removal of minor impurities.

#### 2.5.1 Method Validation

Palytoxin and ovatoxin-a calibration solutions were prepared from 1 to 500 ng/mL and used to verify the linear response of palytoxin by LC–MS/MS (Figure S2). Repeatability was assessed by injecting palytoxin standards at three concentration levels in 5 replicate injections (Table S1).

## 3 Results and Discussion

### 3.1 pH Stability

Palytoxin has previously been shown to undergo acid-catalyzed hydrolysis at low pH.^27^ In order to more accurately determine the pH range associated with palytoxin instability, palytoxin and ovatoxin-a were monitored in 100 mM buffers ranging from pH 2 to 10 and using 100 mM HCl (pH 1) and 100 mM NaOH (pH 13) to represent the effects of strong acid and strong base, resulting in approximate pHs of 1.3 and 12.7 in the final solutions, respectively. For ease of discussion, these will be referred to as pH 1 and 13 in the following results. These were mixed 1:1 with MeOH for diluting palytoxin and ovatoxin-a to assess short-term stability at room temperature over one to 2 days.

A time-dependent decrease in the palytoxin response was observed at the pH extremes of pH 1, pH 2, and pH 13 (Figure 2a). LC–HRMS data acquired on the samples stored under these extreme pH conditions showed degradation of palytoxin (Figure S3). Under acidic conditions, formation of the methyl ester from methanolysis at C-1 of palytoxin was observed with an [M + 2H]^2+^ at *m*/*z* 1283.7216, consistent with Mazzeo et al. (2021).^27^ No degradation products were observed in positive ionization mode by LC–HRMS following complete degradation of palytoxin in strong base. Plots of the palytoxin signal over time indicated pseudo-first-order kinetics, which were plotted with 2-parameter exponential decay curves to estimate the observed half-lives. The half-life was 190 min at pH 1, 2800 min (2.4 days) at pH 2, and 1300 min (21 h) at pH 13. Under these conditions, there was no detectable loss of palytoxin from pH 3 to pH 10 for 2 days at ambient temperature (Figure 2b).

**Figure 2:**
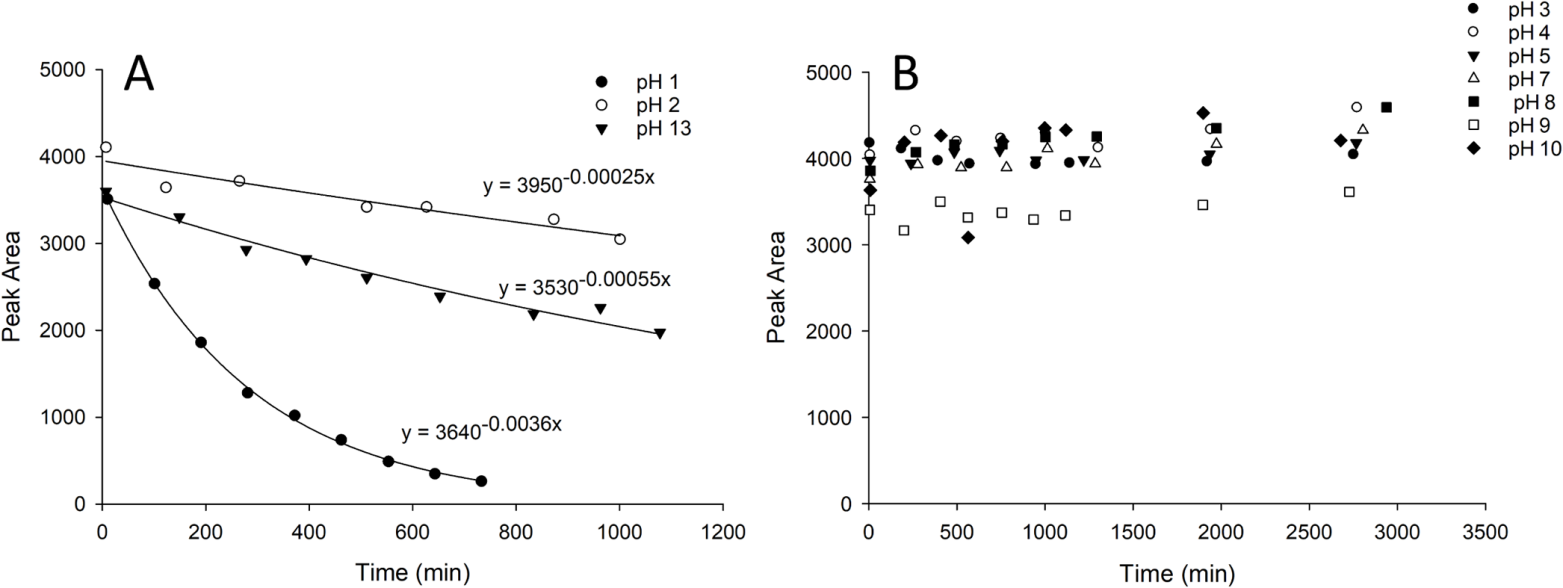
Palytoxin response over time at various pH values when prepared in 1:1 MeOH–buffers at ambient temperature: (A) at pH 1, pH 2, and pH 13; and (B) at pH 3, 4, 5, 7, 8, 9, and 10.

The pH stability of palytoxin was also assessed in a lower percentage of MeOH, at a ratio of 1:9 MeOH–buffer (Figures S4 and S5). The increased aqueous percentage in the solution led to a much faster loss of palytoxin under strongly acidic conditions (pH 1), with a half-life estimated at 82 min. Additionally, in these highly aqueous conditions, palytoxin was less stable at intermediate pH ranges, where detectable loss was observed below pH 5 and above pH 8.

Instability for ovatoxin-a was demonstrated in both strong acid (pH 1), base (pH 13), and at pH 2 (Figure 3a), while no detectable loss of ovatoxin-a occurred between pH 3–10 at ambient temperature for 24 h (Figure 3b). Ovatoxin-a was considered to be more unstable than palytoxin, as evidenced by a shorter half-life in strong acid/base conditions of 8 min at pH 1, 320 min at pH 13, and 3500 min at pH 2.

**Figure 3:**
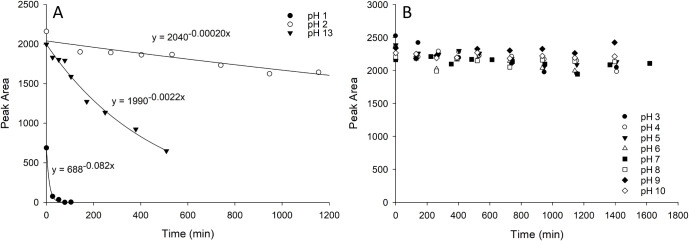
Ovatoxin-a response over time at various pHs when prepared in 1:1 MeOH–buffer and stored at ambient temperature: (A) at pH 1, pH 2, and pH 13, where losses over time were observed, and (B) at pH 3, 4, 5, 6, 7, 8, 9, and 10, where there was no observable loss of ovatoxin-a for the duration of the experiment.

### 3.2 Solvent Composition

The LC–MS peak area of palytoxin in the 1:9 MeOH–buffer solutions (Figure S5) was approximately 70% of that obtained under the control conditions (1:1 MeOH–H_2_O; Figure 2b), suggesting an impact of solvent composition on palytoxin solubility or some other interaction leading to a reduced response. To study this further, a series of dilutions from 0–100% MeOH (at 10% increments) were used to assess palytoxin and ovatoxin-a LC–MS/MS response. The minimum percentage of MeOH required to obtain the expected response was found to be 50%, with the response declining significantly at lower percentages (Figure 4). Preliminary experiments in which the two analogues were tested separately suggested that palytoxin behaved differently from ovatoxin-a in low percentages of MeOH (Figures S6 and S7). Replicate preparations of these samples as mixed solutions of these two analogues indicate that their behavior at low concentrations is similar (Figure S8). Comparison of samples before and after centrifugation showed that, in 0–30% MeOH, both compounds were insoluble in highly aqueous solvents and can be removed from solution by centrifugation (Figure 4). Therefore, the originally observed differences may be due to the inhomogeneity during analysis and the rate of precipitation of the palytoxins.

**Figure 4:**
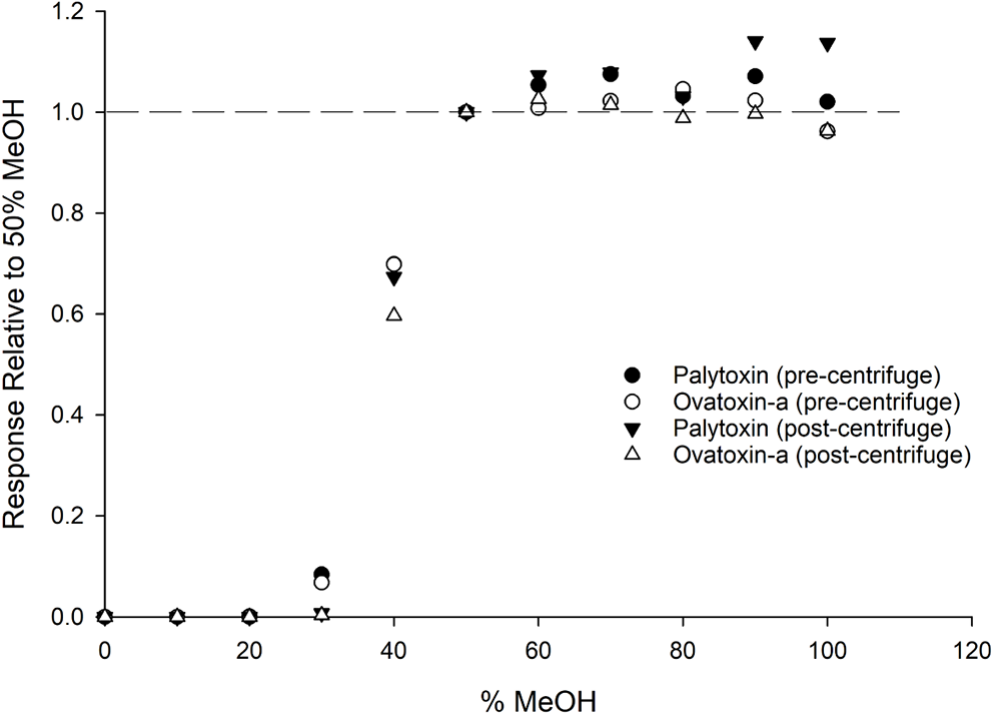
LC–MS/MS peak area response of palytoxin (335 ng/mL) and ovatoxin-a (354 ng/mL), prepared in solvents with varying percentages of MeOH, relative to the response in 50% MeOH (control). An aliquot of each solution was analyzed before and after centrifugation.

The results of this study differ from those by Mazzeo et al. (2021),^27^ which showed a higher palytoxin response in 10–50% aqueous MeOH, while decreasing at higher organic solvent concentrations. This indicates a need to assess organic solvent concentration effects on instrument response before assuming consistency across laboratories. The discrepancy may have arisen from differences in water quality, selected MS transitions/ions, or other uncontrolled sample preparation or instrument response variables.

As noted by Mazzeo et al. (2021),^27^ and confirmed here (Figures 4 and S5–S7), 100% aqueous solvent without the addition of buffer led to nearly complete loss of palytoxin and ovatoxin-a. The exact mechanism of loss is not understood, but similar behavior is also observed in protein and peptide analysis.^30–33^ Given that palytoxin contains one primary amine, two secondary amides, and many hydroxy groups, we hypothesize that under highly aqueous conditions, there is either adsorption to glass, solubility issues, or aggregation that may be occurring that affect analytical measurements.

The centrifugation experiments (Figure 4) suggest the main factor affecting palytoxin and ovatoxin recovery is solubility. A set of palytoxin and ovatoxin-a samples diluted with 1:9 MeOH–H_2_O were prepared and centrifuged, the aqueous solutions removed, and 80% MeOH was added. The objective was to determine whether the toxins were precipitating out of solution. For palytoxin and ovatoxin-a, the recovery in 80% MeOH was 93 and 90%, respectively, compared with the controls (Figure S9), confirming that solubility is the factor reducing recoveries of palytoxins in highly aqueous solutions.

Highly aqueous solutions are often required for analytical, isolation, and toxicological experiments; therefore, the use of additives was assessed to improve the solubility of palytoxins. In peptide analysis, there are a variety of additives that can be used to improve recovery and analysis.^30–33^ BSA and PBS specifically have been previously employed by Poli et al. (2018)^26^ for preparation of aerosolized palytoxin. Several aqueous solutions were prepared to assess the effects of different additives on the solubility and LC–MS/MS responses of palytoxin and ovatoxin-a. For palytoxin, the presence of BSA significantly improved solubility, and the further addition of salts (or ions) such as PBS, phosphate buffer, or NaCl improved recovery to nearly 100% compared to a control in 50% MeOH (Figure 5). For ovatoxin-a, the addition of BSA and other salts/ions improved solubility/recovery, but the recoveries were approximately 50% compared with the control. Differences in the behavior of palytoxin and ovatoxin-a may be due to structural differences between the analogs that could affect their 3-dimensional conformation and solubility. Ovatoxin-a contains a hydroxy group at C-42, whereas palytoxin does not but instead has hydroxy groups at C-17, C-44, and C-64, which could enhance the aqueous solubility of palytoxin relative to ovatoxin-a. These results strongly suggest that the effects in highly aqueous solutions are similar to those observed in protein recovery^30^ and that BSA and ions in solution provide options to improve the recovery and solubility of palytoxin and ovatoxin-a.

**Figure 5:**
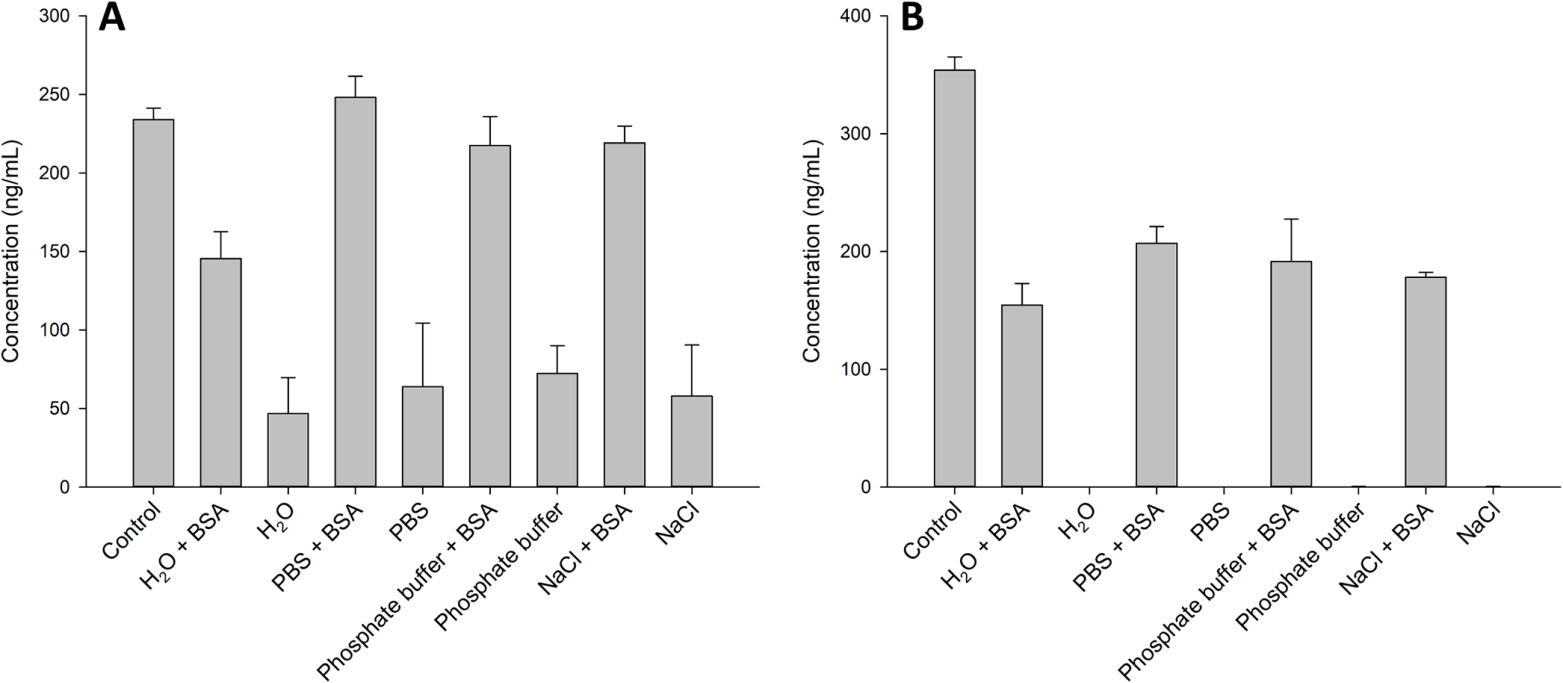
Measured concentrations (LC–MS/MS) of: (A) palytoxin, and; (B) ovatoxin-a, following dilution to highly aqueous conditions with various additives (final solvent composition of 1:9 MeOH–aqueous) compared to the control of 1:1 MeOH–H_2_O.

### 3.3 Evaporation Experiments

Evaporation during palytoxin isolation has been shown to affect recovery.^27,28^ While Mazzeo et al. (2021)^27^ performed preliminary experiments assessing recovery of palytoxin during evaporation from solutions containing <50% water, isolation processes may require evaporation or solvent removal from more highly aqueous solutions. Mazzeo et al. (2021)^27^ also reported that the contact surface can impact palytoxin recovery. To further evaluate this, experiments were performed with nonsilanized glass and polypropylene vials using a variety of evaporation techniques. Solutions were prepared in 10% and 50% MeOH and in 1:9 MeOH–PBS+BSA, evaporated to dryness, and reconstituted in 50% MeOH. In glass, the recovery was low for 10% and 50% MeOH, while the solutions containing PBS and BSA resulted in high recovery of palytoxin (Figure 6). In polypropylene vials, the recoveries of all solutions increased substantially, while the highest recovery was obtained from the PBS + BSA solutions.

**Figure 6:**
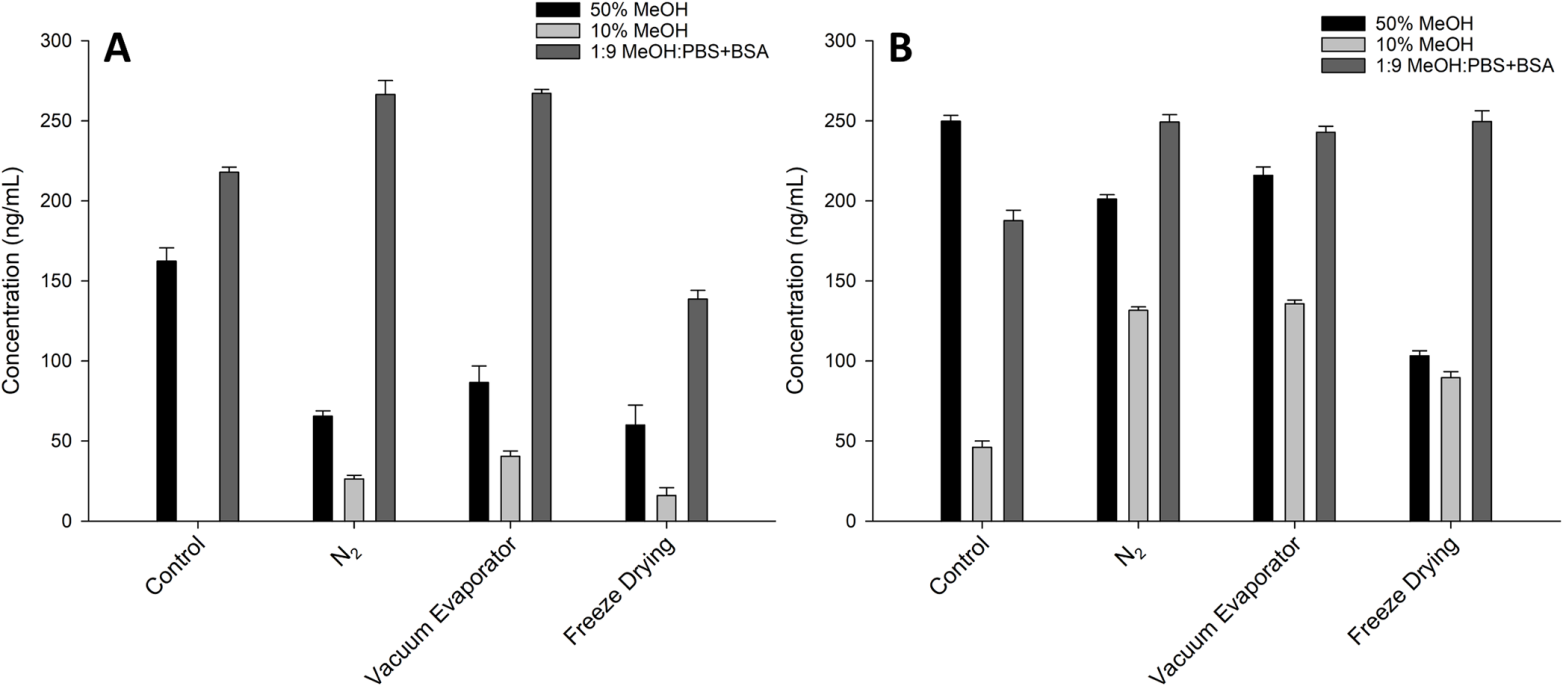
Measured concentrations (LC–MS/MS) of palytoxin following evaporation using different methods from 1:1 MeOH–H_2_O, 1:9 MeOH–H_2_O, and 1:9 MeOH–PBS + BSA in (A) glass and (B) polypropylene vials.

A second experiment was performed in polypropylene tubes, starting with palytoxin in either 50% MeOH or 1:9 MeOH–PBS + BSA. Following evaporation using a gentle stream of N_2_ gas, one sample was reconstituted in water, while the other two residues were reconstituted in 50% MeOH. In all cases, the recovery was nearly 100% (Figure 7), suggesting that the use of polypropylene is suitable for high recovery. However, in cases where the use of polypropylene is undesirable, the addition of PBS and BSA can be used to improve palytoxin recovery from glass surfaces. Additional experiments are needed to evaluate suitable methodologies to remove these additives to obtain purified palytoxin.

**Figure 7:**
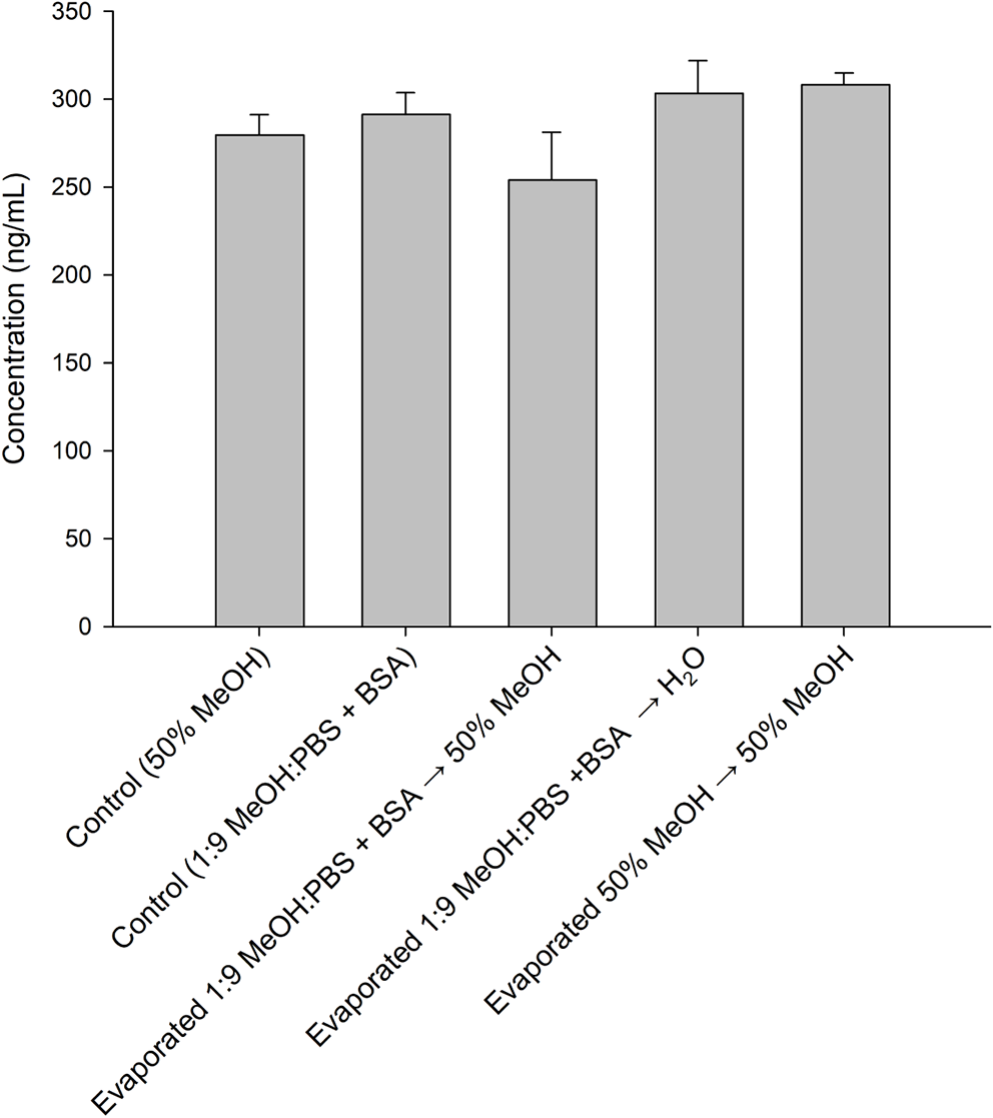
Palytoxin content in either 50% MeOH or aqueous conditions (1:9 MeOH–PBS + BSA) compared to equivalent solutions prepared in these solvents following evaporation under N_2_ and redissolution in either 50% MeOH or water.

Based on these results for palytoxin and ovatoxin-a, it is recommended to maintain solutions with an organic content above 50% MeOH to ensure high recovery and accurate analytical measurements. A neutral pH is recommended, although slightly acidic or basic conditions are suitable for short periods (pH 5–8). If aqueous conditions are necessary, the addition of PBS and BSA will significantly increase recovery. When evaporating solutions of palytoxin, a polypropylene contact surface is preferable to glass, while Mazzeo et al. (2021)^27^ also recommend the use of PTFE. If polypropylene is unavailable or needs to be avoided, then evaporation in glass can be used with the addition of PBS and BSA. Solvent selection for handling palytoxins is critical not only for isolation and analytical measurements but also for toxicological studies. These findings provide guidance for handling palytoxins in various study applications, although confirmation of stability and dissolution should always be verified with novel toxin analogues, solvents, or additives.

While there is clear evidence of acid-catalyzed degradation of palytoxin, the mechanisms underlying other losses during handling are not well understood. In general, the conditions that led to high recoveries were similar for the two analogues, suggesting that solvent selection is similar for different palytoxin analogues. However, some differences in the recoveries of the two analogues were observed under certain conditions. The factors causing these differences could be related to impurities from the isolation procedure (proteins, salts, etc.) or due to structural differences between the two toxins, as described in Section 3.2.

The centrifugation experiments were performed to confirm that the low responses in highly aqueous solutions were due to insolubility. The addition of PBS and BSA appears to be a suitable option for improving the solubility of palytoxin and ovatoxin-a in highly aqueous solutions, similar to the effects observed with proteins and peptides.^34–36^ A variety of surfactants or small amounts of organic solvents (MeCN or DMSO) have also been suitable for protein solubility.^32,37–39^ Therefore, future work could explore suitable additives for solubilizing palytoxin analogues, in addition to the development of protocols for their subsequent removal during isolation protocols.

## 4 Conclusions

The objective of this work was to understand the solubility and establish improved handling protocols for palytoxins, including optimal solvents for dissolution. Given that some differences were observed between palytoxin and ovatoxin-a, consideration of structural differences is necessary when evaluating the handling of individual palytoxin analogues to verify that conditions are appropriate for the analogue being studied. The optimal conditions indicate that palytoxins must be maintained in greater than 50% organic solvent such as methanol at a pH range of 5–8. These conditions can be used to develop improved analytical procedures for accurate quantitation of palytoxin, as well as to ensure high yields during isolation to enable development of reference materials for a variety of analogues that can be used for toxin verification, toxicity evaluations, and environmental monitoring. These results emphasize the importance of properly quantifying toxins based on both their solubility and stability in solutions used for analytical measurements, toxicity evaluations, and complex environmental samples.

## Supplementary Material



## Data Availability

The data underlying this study are available in the published article and its online Supporting Information.

## Notes

LC-MS/MS chromatograms; LC-MS/MS validation data; LC-HRMS methodology and analysis of palytoxin degradation products in unstable conditions; time-dependent monitoring of palytoxin in high aqueous solutions at various pHs; repeated experiments in varying percentages of MeOH; recovery from centrifugation of aqueous preparations (PDF)
