# Supplementary material for: Stability and Recovery of Palytoxin and Ovatoxin‑a
Source: ACS Omega. 2025 Nov 4;10(45):54039–47. doi: 10.1021/acsomega.5c05500 (PMC12631699; doi:10.1021/acsomega.5c05500)
Supplement: Supplementary file 1 [file ao5c05500_si_001.pdf]

Supplemental Information

Stability and Recovery of Palytoxin and Ovatoxin-a

Elizabeth M. Mudge[1*], Christopher O. Miles[1,2], Valentina Miele[3], Carmela Dell'Aversano[3], Pearse McCarron[1]

[1] Metrology Research Centre, National Research Council of Canada, 1411 Oxford Street, Halifax, NS, B3H 3Z1 Canada

[2] Norwegian Veterinary Institute, P.O. Box 64, 1431 Ås, Norway

[3] University of Napoli Federico II, School of Medicine and Surgery, Department of Pharmacy, Via D. Montesano 49, 80131 Napoli, Italy

Corresponding Author:

Elizabeth M. Mudge

National Research Council Canada

1411 Oxford Street, Halifax, NS Canada

Email: elizabeth.mudge@nrc-cnrc.gc.ca

Phone: +1-902-426-6458

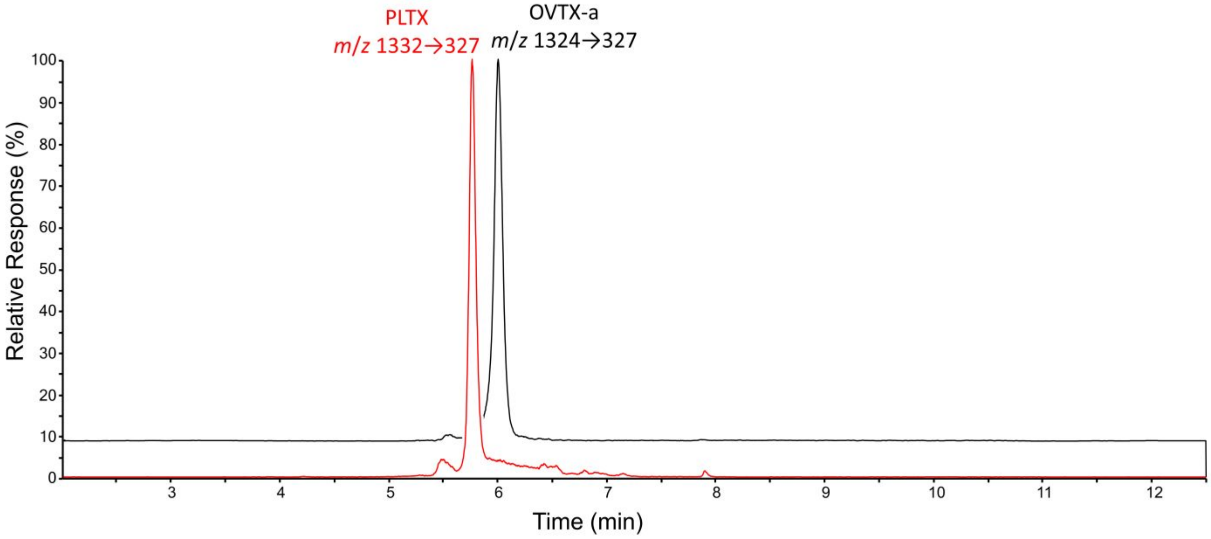

Figure S1. LC-MS/MS chromatogram of palytoxin (red; SRM transition *m*/*z* 1332→327) and ovatoxin-a (black; SRM transition *m*/*z* 1324→327).

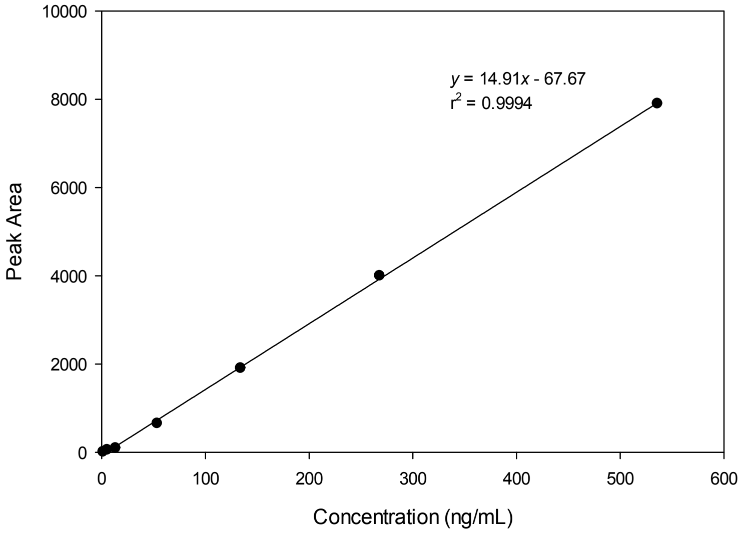

Figure S2. Calibration curve for palytoxin from 1 to 500 ng/mL.

Table S1. Instrument repeatability between five replicate injections of palytoxin at three different concentrations.

Concentration (μM)	Average Peak Area	RSD (%)
0.50	14640	2.1
0.25	7058	2.2
0.05	1084	5.7

LC-HRMS analysis of strong acid and base stability samples of palytoxin

An Agilent 1290 Infinity II LC equipped with a binary pump, temperature-controlled autosampler (10 °C) and column compartment (25 °C) (Agilent Technologies, Mississauga, ON, Canada) coupled to a Q Exactive HF Orbitrap mass spectrometer (ThermoFischer Scientific) with a heated electrospray ionization probe (HESI-II). Chromatographic separation was achieved using a Kinetex C18 column (Phenomenex, 100 × 2.1 mm, 2.6 µm) using gradient elution with mobile phases composed of 30 mM acetic acid in both H_2O (A) and 95:5 MeCN:H_2O (B). The flow rate and injection volumes were 0.4 mL/min and 5 µL, respectively. The gradient was: 0–7 min, 23–32% B; 7–7.5 min, 32–100% B; 7.5–10 min, 100% B; followed by a 2.5 min re-equilibration at 23% B. Full-scan acquisition was performed from *m/z* 800–1400 in positive mode. The spray voltage of the source was +4.8 kV, with a capillary temperature of 275 °C. The sheath and auxiliary gas were set at 38 and 10 (arbitrary units). The auxiliary gas heater temperature was set at 300 °C and the S-Lens RF level was set to 100. The mass resolution setting was 60 000 with an automatic gain control (AGC) target of 5×10^6 and a maximum injection time of 200 ms per scan.

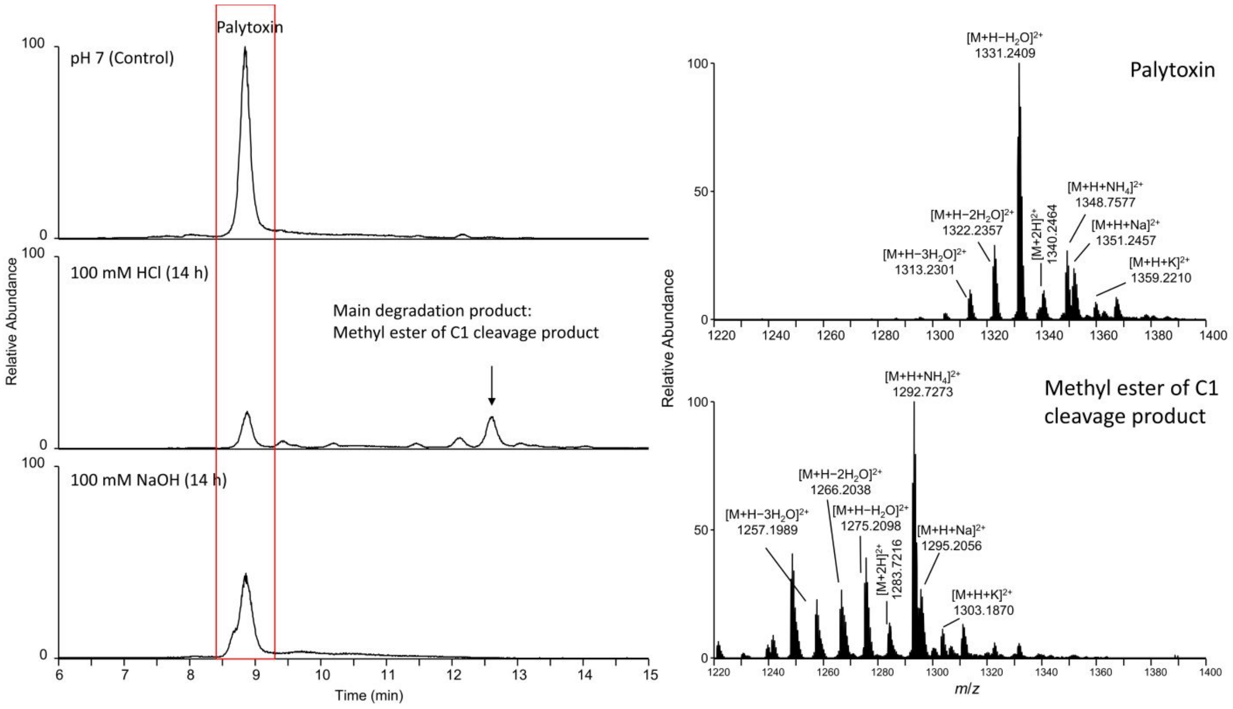

Figure S3. (Left) LC–HRMS base-peak chromatograms of palytoxin prepared in 50% MeOH containing 100 mM phosphate buffer pH 7 (top), 100 mM HCl (middle) and 100 mM NaOH (bottom) as the aqueous diluent after 14 h at ambient temperature. (Right) HRMS full scan mass spectra of palytoxin (top) and the main degradation product observed in 100 mM HCl which is the methyl ester of C1 cleavage of palytoxin (bottom).

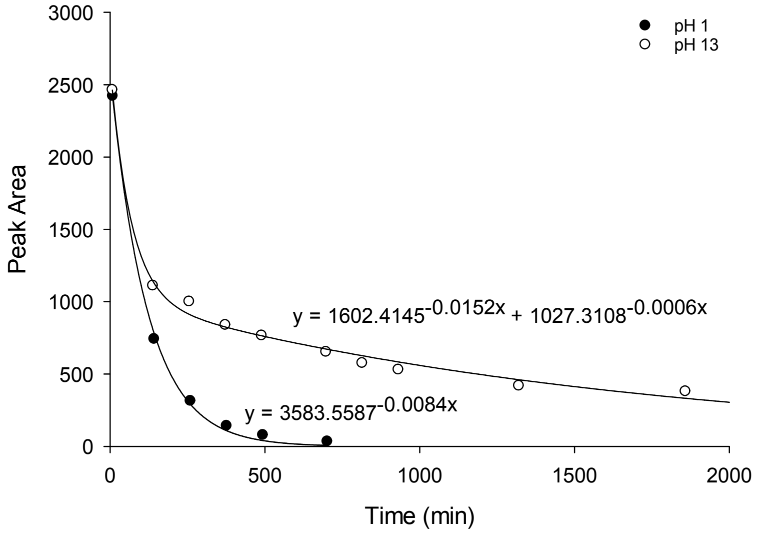

Figure S4. The peak area response for palytoxin in strong acid and base conditions by dissolving in 1:9 MeOH:H_2O containing 100 mM HCl and 100 mM NaOH, respectively.

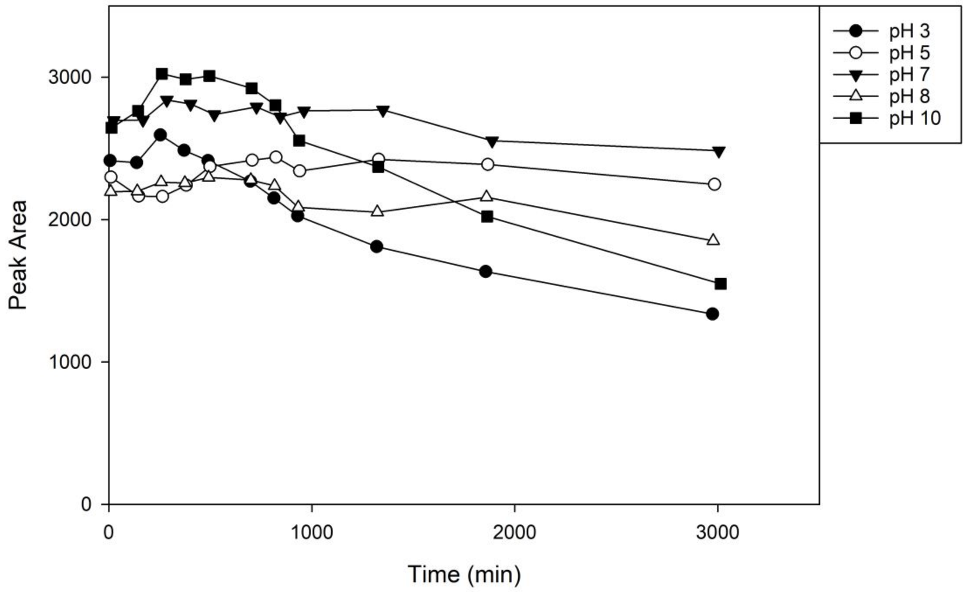

Figure S5. The peak area response for palytoxin with time after diluting to 1:9 MeOH:H_2O containing 100 mM buffers from pH 3 to 10.

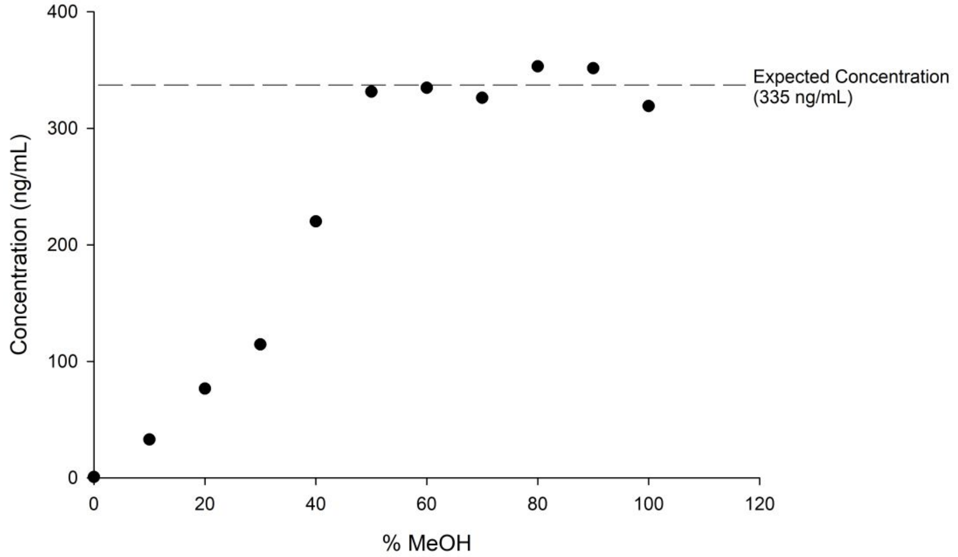

Figure S6. Measured concentrations of palytoxin, based on LC–MS/MS response, prepared in solvents with varying percentages of MeOH, with the expected concentration of 335 ng/mL marked as a dashed line.

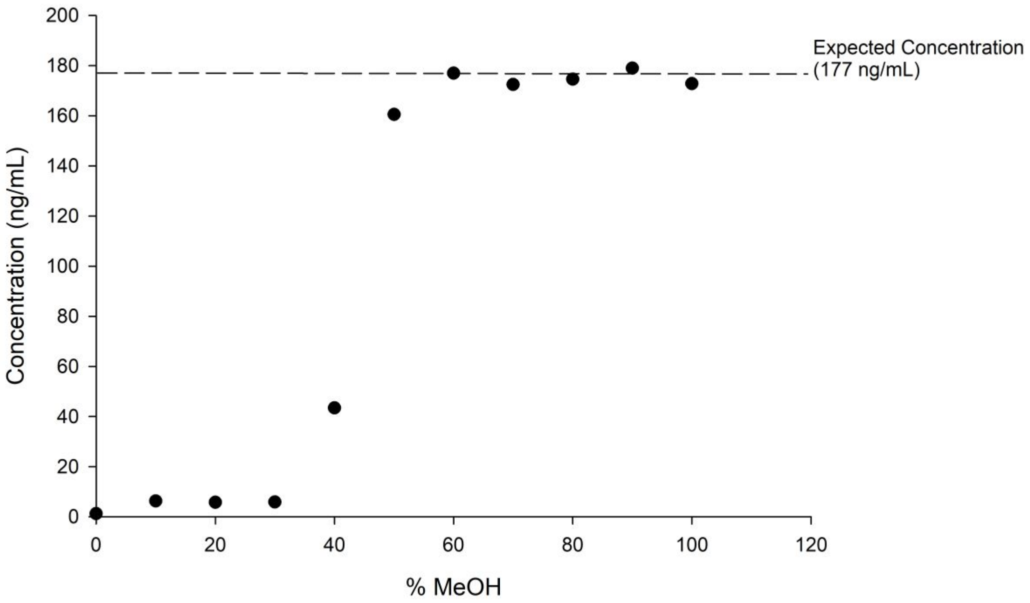

Figure S7. Measured concentration of ovatoxin-a, based on LC–MS/MS response, prepared in solvents with varying percentages of MeOH, with the expected concentration of 177 ng/mL marked as a dashed line.

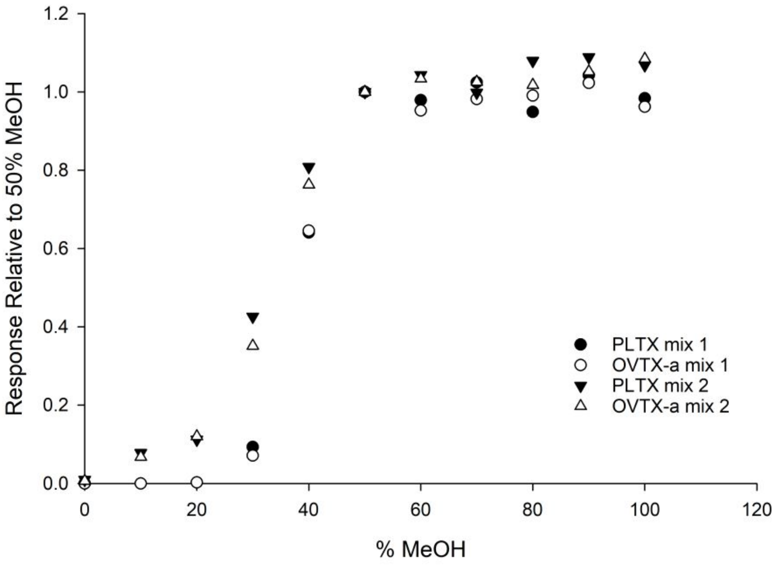

Figure S8. LC–MS/MS peak area response of palytoxin and ovatoxin-a mixtures prepared two separate days (mix 1, mix 2) in solvents with varying percentages of MeOH, relative to the response in 50% MeOH (control).

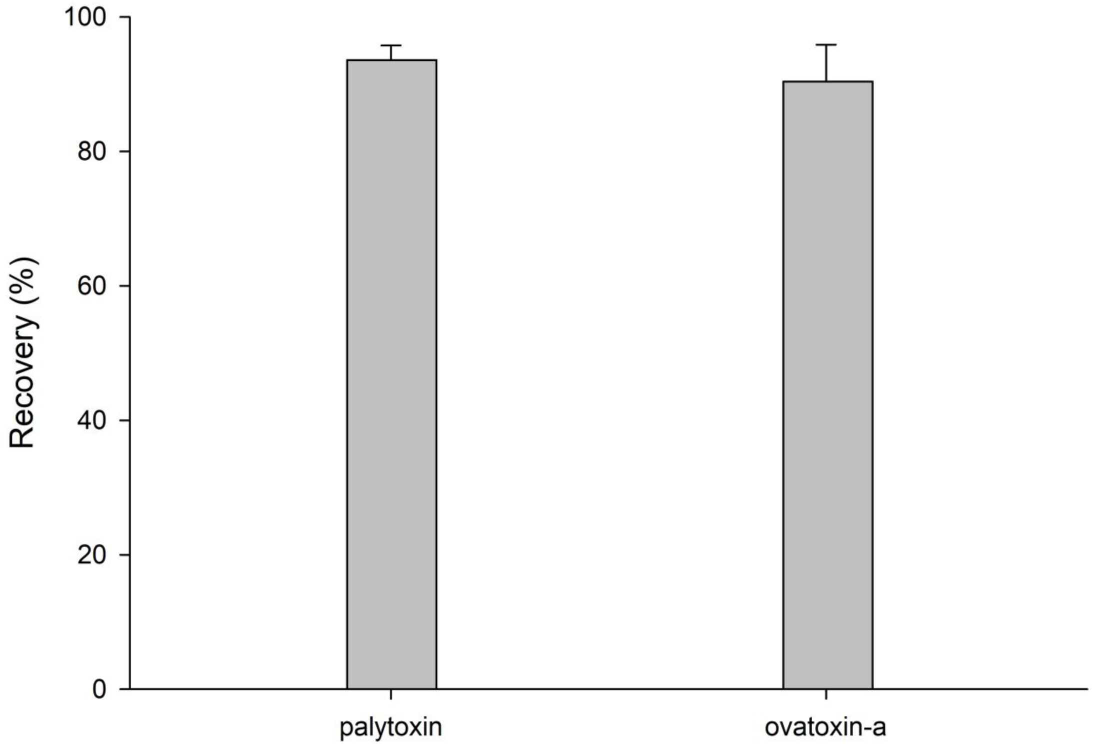

Figure S9. Palytoxin and ovatoxin-a recovery following centrifugation of aqueous preparations containing the two analogues, separately. The aqueous solution was removed and 80% MeOH was added to assess recovery against controls prepared at the same concentrations.
